# The stress response of intensive care unit medical doctors facing repeated severe emergencies

**DOI:** 10.3389/fpsyg.2022.895954

**Published:** 2022-11-24

**Authors:** Roberta Ciuffini, Alfonso Marrelli, Cinzia Leuter, Paolo Stratta, Antonella Paladini, Alessandra Ciccozzi, Ida Marsili, Franco Marinangeli, Alba Piroli

**Affiliations:** ^1^Department of Life, Health and Environmental Sciences, University of L’Aquila, L’Aquila, Italy; ^2^San Salvatore Hospital, L’Aquila, Italy; ^3^Campus Bio-Medico University, Rome, Lazio, Italy; ^4^ASL 1, Department of Mental Health, L’Aquila, Italy

**Keywords:** COVID-19, intensive care unit (ICU) doctors, work functioning, stress reactions, repeated trauma exposure

## Abstract

**Objectives:**

This study assesses the psychopathological distress experienced by doctors working in an Intensive care unit (ICU) during the COVID-19 pandemic. These doctors were the same who faced the consequences of a previous natural disaster, a severe 6.3 magnitude earthquake. A second objective is to evaluate their current mental attitude, professional performances and coping strategies adopted in the pandemic in relation to the conditioning effect of that first emergency, the earthquake.

**Methods:**

Thirty-seven ICU medical doctors were recruited and assessed using Rapid Stress Assessment (RSA) rating scale, Symptom Checklist-90 Revised (SCL-90-R), Zung Self-Rating Anxiety Scale, Beck Depression Inventory, Beck Hopelessness Scale, Millon Clinical Multiaxial Inventory III. Comparison between exposure to the earthquake and COVID pandemic has been made in terms of professional role and psychological burden.

**Results:**

Comparison between 2009 earthquake catastrophe and COVID pandemic conditions evidenced relevant changes in professional role, team, environment, shifts, and work organization.

**Conclusion:**

The doctors, who already experienced the 2009 earthquake reported a feeling of greater insecurity facing this latter catastrophe, the COVID pandemic, as well as perception of greater concern for their family and the global situation. However, having participated in the medical management of another emergency (the 2009 earthquake) appears to have contributed to limiting demoralization and psychological distress. The feeling of having greater decision-making possibilities and participation in the organization of work, strengthen coping skills in the face of the emergency.

## Introduction

Several studies have shown how natural disasters and global health criticisms cause a significant increase in stress factors with significant physical and mental repercussions (Ripoll Gallardo et al., 2018; Maciaszek et al., 2020). The medical profession has one of the highest rates of work-related stress (Shanafelt et al., 2012) with a particularly high incidence among professionals working in the intensive care units (ICU) (Andrade Oliveira and Andrade Dantas, 2015; Embriaco et al., 2016). The various stressors, associated with the type of work situations, can be a source of concern and can give rise to significant consequences that often impact on the professional’s family life, with less job satisfaction, anxiety, depression, insomnia, alcohol or drug abuse, and a tendency to make more mistakes (Kushal et al., 2018; Adams and Walls, 2020). Healthcare professionals are involved on two levels: First, they experience the impact the event has on everyone, while at the same time their workload necessarily becomes more demanding and intense. This translates into a further increase in stress factors (Mattei et al., 2017) due to the need to provide emergency aid in a climate of general uncertainty.

Pandemic such as COVID-19 cause to the healthcare professionals caring for infected patients, a great deal of anxiety and stress due to the fear of contracting the disease or passing it on to their families. It is important to examine and monitor the psychological characteristics of the healthcare professionals involved, to address the potential risk of burnout in these circumstances. Resilience seems to be the key to investigating the two opposite profiles (low/high) at risk of burnout according to the recent study carried out by Di Trani et al. (2021) whose results show that the levels of individual resilience and one’s ability to tolerating uncertainty were significant factors in determining the impact of the COVID-19 emergency on healthcare workers. Furthermore, the use of emotional strategies that allow people to remain in a critical situation without the need to control it seems to protect against burnout (Di Trani et al., 2021).

Burnout syndrome has a profound effect on the mental health status of healthcare workers. The most frequent risk factors associated with it include stress, lack of family support, and organize the work such as prolonged night shifts, length of experience, and exposure to traumatic events. Individuals and the hospital authority need to pay specific attention to work-related stress risk factors to improve the psychological wellbeing of healthcare workers (Magnavita et al., 2021).

Furthermore, the lack of healthcare resources and the growing number of suspected and/or confirmed cases add to the pressures and concerns felt by healthcare professionals (Buselli et al., 2020; Neto et al., 2020; Quintana-Domeque et al., 2021).

The COVID-19 outbreak has placed extraordinary demands on health systems worldwide, putting a strain on the wellbeing of healthcare workers. Emergency medicine is characterized by a highly emotionally charged process of caring for critically ill patients, and doctors who operate on it are more prone to developing burnout and post-traumatic stress disorder (PTSD), either due to emotional trauma or due to the cumulative stress on the practice.

A significant proportion (35%) in the Northeastern United States experienced acute post-traumatic symptoms during the COVID-19 pandemic crisis, potentially indicating a high prevalence of acute stress disorder and an increased risk of developing PTSD. Thus, early detection of physicians at risk and referral to assessment and treatment may be important for mitigating pandemic-related PTSD (Chang et al., 2022).

The main objective of this study is to evaluate the psychopathological distress experienced by the intensive care doctors of the San Salvatore Hospital of L’Aquila (Italy) during the COVID-19 pandemic.

L’Aquila is the town hit on 6 April 2009, by a 6.3 magnitude earthquake. Three hundred nine people died, 1,600 were injured, 65,000 were evacuated and 11,000 homes were damaged. The ICU doctors of the San Salvatore Hospital have been heavily involved in the rescue of seriously injured people. The doctors facing the current pandemic are therefore the same doctors who have faced the consequences of that natural disaster.

The intense, confused, and frightening emotions that follow a traumatic event can be even more pronounced in people directly and repeatedly exposed to traumatic stress (Brewin et al., 2017). Repeated exposure to disasters can determine differences in stress adaptation and resilience with consequences on the efficiency of emergency management (Stratta et al., 2021).

The second objective is therefore to verify the mental attitude, professional performances, and coping strategies adopted in relation to the conditioning effect that the first emergency, the 2009 earthquake, may have caused on the second, the current pandemic.

## Materials and methods

### Participants

Participants in the study were 37 medical doctors (31 females and 6 males) working at the S. Salvatore Hospital ICU: Mean age of 41.5 years (SD 11.6), years of service 13.2 (2.8 SD). All the recruited doctors were specialized in Anesthesiology, Reanimation and Intensive Care and they had been present and on duty in the aftermath of the L’Aquila 2009 earthquake.

### Procedure

A cross-sectional observational study was designed and carried out between 1 February and 1 March 2020, during the second “wave” of the Italian COVID-19 pandemic phase 1 when the health workers experienced the greatest work-related stress burden.

Each participant was given a questionnaire concerning sociodemographic, occupational data, and rating scales to be fulfilled in complete anonymity.

### Measures

Rapid Stress Assessment (RSA) (Tarsitani and Biondi, 1999) is an instrument designed to self-assess the perception of the effects of acute and chronic stress, appropriate for use in studies involving very large samples also. This scale was used due to a lack of validated Italian versions of short psychometric instruments to evaluate subjective stress in large samples. The scale explores the individual response to stressful situations as it is specific, flexible, convenient, and easy to administer and can be sufficiently reliable and valid (Pancheri et al., 2002).

The rating scale is composed of 15 items on a 4-point likert scale; it divides stress assessment into five areas, each evaluated by three items (range 0–9): Anxiety, depression, somatization, aggressiveness, and social support. Social support is not a psychopathological dimension but is considered an essential factor in the response to stressors and was assessed as a negative scale quantifying the lack of support. The sum of all items (range 0–45) gives a total stress score, with higher score reflecting a higher perception of emotional distress.

*Symptom Checklist-90 Revised (SCL-90-R)* (Derogatis, 1994) is a 90-item self-report instrument designed to assess mental health symptoms across nine subscales (somatization, obsessive-compulsive disorder, interpersonal sensibility, depression, anxiety, hostility, phobic anxiety, paranoid ideation, and psychoticism) and three global scales. Respondents are asked to rate the severity of their symptoms on a scale of 0 to 4 (0 = not at all to 4 = extreme). The three global scales are: the Global Severity Index (GSI), indicator of mental stress intensity; the Positive Symptom Total (PST), which represents the number of symptoms reported per subject; the Positive Symptom Distress Index (PSDI), used as an index of the response style, i.e., to what extent the subjects accentuate or minimize their distress.

It was originally conceived to reflect the psychological symptom patterns of psychiatric patients and “non-patients,” with four main aims: (I) To identify symptoms in apparently normal subjects; (II) assess any specific or overall changes in symptoms; (III) to form the basis for clinical predictions and prognoses; (IV) assist clinicians in making a diagnosis in accordance with the Diagnostic and Statistical Manual of Mental Disorders (DSM) (American Psychiatric Association [APA], 2000). Derogatis (2017) published a full review of the criteria-based validity studies on the Brief Symptom Inventory (BSI). The GSI represents the most sensitive single quantitative indicator concerning the respondent’s overall psychological distress status on the SCL-90-R/BSI/BSI-18 series of tests. The SCL-90-R and BSI have also been utilized effectively in treatment planning studies with a healthcare systems orientation (Derogatis, 2017).

*Zung Self-Rating Anxiety Scale (SAS)* (Zung, 1971) is a 20 items self-report scale designed to assess a variety of anxiety symptoms, both psychological and somatic, in adults not already receiving treatment. Responses are given on a 4-point scale ranging from 1 (none, or a little of the time) to 4 (most, or all the time). The scores are grouped in four ranges: 0–20: Very low anxiety, 21–40: Low anxiety, 41–60 moderate anxiety, 61–80: High anxiety. Conti (1999) translated the Italian version of the SAS. The author of the Italian version does not provide data regarding the methodology used for the adaptation from the American original, nor bibliographical references to trace such data; therefore, it is not possible to assess whether adequate standards have been followed to ensure the semantic equivalence of the text (Bruzzi et al., 2004). In any case, the Zung scale obtained a satisfactory reliability value (α = 0.75) regarding the correlation indices that can be superimposed on the competing scales for measuring anxiety.

*Beck Depression Inventory (BDI-II)* (Montano and Flebus, 2006). The BDI-II is an improvement and a renovation of the first edition of Beck Depression Inventory (BDI) (Beck et al., 1961) and of his partial revision, according to DSM-IV diagnostic standards, administered, and validated on an Italian population. Is a self-statement of 21-item for evaluating the severity of depression in normal and psychiatric populations. Each item answer is scored on a scale value of 0–3. Higher total scores indicate more severe depressive symptoms. The standardized cut-offs are: 0–13: Minimal depression, 14–19: Mild depression, 20–28: Moderate depression, and 29–63: Severe depression.

*Beck Hopelessness Scale (BHS)* (Beck et al., 1974). The Italian validated version of the Beck Hopelessness Scales (BHS) is a self-administered scale used to measure “hopelessness,” defined as a set of negative expectations regarding oneself and one’s future life. It is considered a valid measure to predict suicide ideation regardless of the depressive symptomatology (Ciuffini et al., 2019). It consists of 20 true–false statements scored 1 or 0; the total score is the sum of the individual item scores (range: 0–20). Standardized cut-offs group “hopelessness” in four ranges: 0–3: None, 4–8: Mild, 9–14: Moderate, 15 or above: Severe. A score equal to or greater than nine on the scale is considered indicative of a significant suicidal risk.

*Millon Clinical Multiaxial Inventory III (MCMI-III)* (Millon et al., 1997). It is a self-psychometric test whose great advantage, compared to other tests, is to produce a description using the criteria of the DSM-IV, the most widespread international classification system for psychiatric diseases. This minimizes margins of error during evaluation (Millon et al., 2006).

It is the best tool for making intercultural comparisons because it offers constructive equivalence as the foundation of any intercultural assessment that intends to produce comparative data of target subjects from different cultures (Van de Vijver and Tanzer, 2004).

The study conducted by Pignolo et al. (2017) aimed to explore the factorial structure of the Italian version of MCMI-III by comparing the typicality and congruence between Italian culture and different cultures (Dutch and American). Is a self-report personality and diagnostic inventory, designed to assess 14 personality disorders and 10 clinical syndromes. It is a 175-item, true-false, questionnaire that assesses Axis I (10 clinical syndromes), and Axis II (14 personality disorders) based on the *Diagnostic and Statistical Manual of Mental Disorders* (American Psychiatric Association [APA], 2013). A base rate greater than 75 was considered the cut-off.

### Comparison between exposure to 2009 earthquake and COVID pandemic measures

Information was collected about the participants exposure to the 2009 L’Aquila earthquake. The professional role, i.e., being an Anesthetist or Resuscitation Specialist, during the earthquake and the COVID pandemic was assessed. Questions were asked about changes in the team and work environment, as well as hourly commitment and shifts organization.

The comparison of the psychological burden between the 2009 earthquake and the COVID-19 pandemic was also evaluated in terms of Perception of environmental safety, Concern for oneself, Concern for one’s family, Concern for the global situation, Participation in the organization of work, Commitment and Ability to deal with the emergency.

### Statistical analysis

Descriptive statistics were provided for the sample variables. Categorical variables were described by frequency and percentage, continuous variables by mean, and standard deviation (SD).

To avoid the normality issues in the data set non-parametric statistic was chosen. The differences in scores were evaluated by stratifying the sample according to the presence/absence of participation in work organization and changes in role within the department due to the emergency using Wilcoxon–Mann–Whitney.

Associations between the Rapid Stress Assessment (RSA) test and the psychometric scales used in this study were measured using the Spearman correlation factor (rho).

Data were analyzed using the Statistical Package for Social Sciences (SPSS^®^, version 24, IBM, USA).

All the statistical tests were two-tailed, and significant alpha value was set at *p* < 0.05.

## Results

The characteristics of the sample at the psychological assessment are shown in Table 1. All the recruited doctors were present at the 2009 earthquake.

**TABLE 1:** Psychological assessment of intensive care unit (ICU) medical doctors (*n* = 37).

Rating scale	Subscales/Scoring	Cut-off	Frequency	
			*N* *	%
***Rapid stress assessment (RSA)***	Anxiety	0–3	16	43.22
		4–6	18	48.6
		7–9	3	8.1
	Depression	0–3	16	43.22
		4–6	18	48.6
		7–9	3	8.1
	Somatization	0–3	16	43.2
		4–6	19	51.4
		7–9	2	5.4
	Aggression	0–3	23	62.2
		4–6	10	27.0
		7–9	4	10.8
	Social support	0–3	25	67.6
		4–6	9	24.3
		7–9	3	8.1
	Total	0–15	12	32.4
		16–30	23	62.2
		31–45	2	5.4
***Symptom checklist-90 revised (SCL-90-R)***	Somatization	≥0.75	25	69.4
	Interpersonal sensibility	≥0.78	8	22.2
	Depression	≥1.0	7	27.0
	Anxiety	≥0.8	7	19.4
	Hostility	≥0.67	8	22.2
	Phobia	≥0.29	10	27.8
	Paranoia	≥0.83	10	27.8
	Psychoticism	≥0.6	7	19.4
	Positive symptom distress index (PSDI)	≥0.86	27	75.0
***Zung self- rating anxiety scale (SAS)***	Very low	0–20	–	–
	Low	21–40	28	75.7
	Moderate	41–60	9	24.3
	High	61–80	–	–
***Beck depression inventory (BDI)***	Minimal	0–13	35	94.6
	Mild	14–19	2	5.4
	Moderate	20–29	–	–
	Severe	30–39	–	–
***Beck hopelessness scale (BHS)***	None	0–3	18	48.6
	Mild	4–8	8	21.6
	Moderate	9–14	2	5.4
	Severe	>15	9	24.3
***Millon clinical multiaxial inventory III (MCMI-III)***	Histrionic personality pattern	>85	4	10.8
	Obsessive-compulsive clinical personality pattern	>75–85	6	16.2
		>85	12	32.4
		>75–85	7	18.9

*N = number of subjects.

At the Rapid Stress Assessment, 62.2% of subjects reported a score indicative of moderate stress, 5.4% indicative of high stress, while 32.4% gave responses consistent with a low stress level (total score mean + SD = 18.95 + 5.87). The areas with the highest scores are anxiety, depression, and somatization.

At SCL-90 evaluation, 75% of the sample showed a PSDI score above the normal cut-off, 69.4% of the somatization scale, 27.8% for the phobia and paranoia scales, 27% for the depression scale, 22.2% for the interpersonal sensitivity and hostility scales, and 19.4% for the anxiety and psychoticism scales. No anomalies in GSI or PST scores were observed.

The SAS revealed that anxiety levels were low in 75.7% of subjects and moderate in 24.3%. The BDI found no significant levels of depression, while the BHS identified demoralization/hopelessness in more than half the respondents (51.4%), which was severe in almost a quarter (24.3%).

The MCMI-III identified high BR scores (>85 and 75–85) in 10.8 and 16.2% of subjects, respectively, for the histrionic personality pattern, and in 32.4 and 18.9% for the obsessive-compulsive clinical personality pattern.

The correlations between the RSA and the psychological assessment are reported in Table 2. Moderately strong correlations are seen with SCL-90 Anxiety and GSI. Strong correlations are observed with Hostility, Mania, and Sleep. Strong correlations are also seen with for SAS, BDI, and MCMIII major depression and delusional disorders scales. Moderately strong correlations are seen with SCL 90 R Somatization, Depression and Paranoia scales and MCMIII depression scale.

**TABLE 2:** Correlation coefficients (Spearman rho) between RSA and psychological assessment.

Rating scale	Subscales	rho	*P-value*
***Symptom checklist-90 revised (SCL-90-R)***	Somatization	0.35	0.03
	Depression	0.36	0.03
	Anxiety	0.51	0.005
	Hostility	0.70	0.005
	Paranoia	0.39	0.02
	Mania	0.65	0.005
	Sleep	0.67	0.005
	GSI	0.46	0.04
***Zung self-rating anxiety scale (SAS)***		0.54	0.005
***Beck depression inventory (BDI)***		0.54	0.01
***Millon clinical multiaxial inventory III (MCMI-III)***	Depression	0.37	0.02
	Major depression	0.53	0.005
	Delusional disorder	0.44	0.01

Only correlations with *p*-value < 0.05 are reported.

Investigating differences between 2009 work condition and organization during the earthquake and the current one during pandemic several changes have been observed (Tables 3A). The change of role (anesthesiologist or resuscitation specialist) because of the COVID-19 pandemic emerges. Resuscitation specialists saw an increase from 29.7 to 56.8%, while anesthesiologist numbers fell from 70.3 to 43.2%. More than half of the professionals (56.8%) changed the work environment or team. The COVID-19 emergency required an increase in working hours (35.1%), The organization of shifts worsened to 54.1%.

**TABLE 3:** Comparison between 2009 earthquake and 2020 pandemic conditions of intensive care unit (ICU) medical doctors (*n* = 37).

(A) Work condition and organization		
Item	*N* *	%
***Professional role in the 2009 earthquake***		
Anesthesiologist	26	70.3
Resuscitation specialist	11	29.7
***Professional role in the 2020 COVID-19 pandemic***		
Anesthesiologist	16	43.2
Resuscitation specialist	21	56.8
***Team changed***		
Yes	16	43.2
No	21	56.8
***Work environment changed***		
Yes	16	43.2
No	21	56.8
***Hourly commitment***		
Increased	13	35.1
Equal	21	56.8
Decreased	3	8.1
***Organization of shifts***		
Worsened	20	54.1
Equal	2	5.4
Improved	15	40.5

**(B) Psychological burden perception**		

**Item**	*N* *	**%**

***Perception of environmental safety***		
Increased	8	21.6
Equal	12	32.4
Decreased	17	45.9
***Self-concern***		
Increased	20	54.1
Equal	17	45.9
Decreased		
***Concern for one’s family***		
Increased	32	86.5
Equal	5	13.5
Decreased		
***Concern for global situation***		
Increased	33	89.2
Equal	4	10.8
Decreased		
*Participation in the organization of work*		
Increased	22	59.5
Equal	4	10.8
Decreased	11	29.7
***Commitment***		
Increased	21	56.8
Equal	8	21.6
Decreased	8	21.6
***Ability to deal with the emergency***		
Increased	32	86.5
Equal	5	13.5
Decreased		

*N = number of subjects.

Table 3B shows the psychological burden perception comparison between the 2009 and 2020 emergencies. A total of 45.9% of the sample feel lower personal safety in the COVID-19 pandemic. A total of 54.1% feel more self-concern, 86.5% more concern for own family, and 89.2% for global situation. A total of 59.5% of the ICU doctors consider feel their participation in the organization of work increased with respect to the earthquake, 10.8% equal while 29.7 less involved. A total of 56.8% refers increased commitment and 86.5% more ability to deal with the COVID-19 than 2009 earthquake emergency.

To evaluate the association between psychological distress and certain working conditions, comparisons between different participation in the organization of work at the earthquake and at the pandemic have been made (Table 4). Doctors feeling their participation in the organization of work increased at the actual pandemic, reported less distress as evaluated by RSA for the scores pertaining to the “Somatization” and “Aggression” scales as well as Total score. Lower scores in the Hostility, Paranoia, Mania, Sleep, and GSI scales of the SCL-90-R and in the BDI have been also observed.

**TABLE 4:** Psychological distress comparisons between different participation in the organization of work at the earthquake and at the pandemic of intensive care unit (ICU) medical doctors (*n* = 37).

Participation in the organization of work				
Rating scale	Total (*n* = 37)	Equal/Increased (*n* = 22)	Decreased (*n* = 15)	*Wilcoxon–Mann–Whitney test (P-value)*
**Rapid stress assessment (RSA)**				
Somatization	3.4 ± 2.1	2.7 ± 1.7	4.5 ± 2.4	0.04
Aggression	2.8 ± 2.3	1.8 ± 1.6	4.5 ± 2.3	0.005
Total	18.9 ± 5.9	16.7 ± 2.9	22.7 ± 7.1	0.02
***Symptom Checklist-90 Revised* (SCL-90-R)**				
Hostility	0.39 ± 0.4	0.25 ± 0.3	0.60 ± 0.5	0.01
Paranoia	0.59 ± 0.7	0.46 ± 0.6	0.83 ± 0.8	0.03
Mania	0.62 ± 0.6	0.42 ± 0.4	0.94 ± 0.8	0.01
Sleep	0.81 ± 0.9	0.46 ± 0.5	1.3 ± 1.1	0.01
GSI	0.50 ± 0.6	0.37 ± 0.4	0.73 ± 0.7	0.03
***Beck Depression Inventory* (BDI)**	4.8 ± 4.6	3.5 ± 4.0	6.7 ± 4.9	0.02

## Discussion

This study has the dual aim of investigating the mental state of ICU doctors facing critical working conditions during the COVID pandemic crisis and the possible influence of having faced another, albeit different, natural catastrophe, namely the 2009 L’Aquila earthquake.

The COVID pandemic lead to focus relevant attention to importance of healthcare providers, their burden working on the frontlines and therefore the consequences to their mental health in terms of experience of emotional distress (Carmassi et al., 2021; Ghahramani et al., 2021; Koontalay et al., 2021; Marcomini et al., 2021; Arnold-Forster et al., 2022). This is furthermore particularly true for healthcare workers primarily involved such as ICU doctors, particularly at risk for their role and kind of work (Verma et al., 2015; Shinde et al., 2020).

The Rapid Stress Assessment (RSA) rating scale we used, a specific instrument to measure the subjective perception of the damage caused by a stressor, identified a significant level of stress in about two-thirds of this sample of ICU doctors, indicating a generic state of alarm and psychological distress.

The RSA individual subscales revealed relevant percentages of professional, about 60%, reporting perception of stress in terms of anxiety, depression, and somatization. The SCL-90-R evaluation, although no significant anomalies in individual scales were observed, evidenced a PSDI score above the cut-off in 75% of subjects.

The RSA subscales strongly correlated with the other rating scales, SCL-90 anxiety, hostility, mania and sleep, SAS anxiety, and BDI depression. This result is coherent with the pivotal content validity study reporting similar correlation with scales of the Minnesota Multiphasic Personality Inventory (MMPI) (Tarsitani and Biondi, 1999).

The stress perception as evaluated by the RSA is based on psychological characteristics and on acute symptoms, reactive to contingent situations, advocating the possible use of this flexible, repeatable, and rapid test as a screening tool and for the longitudinal assessment of wellbeing/malaise.

A reciprocal relationship between reaction to stress and the *de novo* development or exacerbation of mental symptoms, particularly symptoms of anxiety or demoralization, is conceivable. In this regard, knowledge of the endogenous individual psychobiological characteristics is important. Data from the MCMI-III revealed personality patterns and clinical scale scores that may play a favorable or aggravating role in the emotional and stressful burden of working in a health crisis.

Tarchi et al. (2022) examined the prevalence and individual predictors of mental distress among a sample of volunteers participating in emergency services. Personality factors were important predictors for all dimensions (depression, anxiety disorders, emotional exhaustion, depersonalization, and low personal accomplishment).

When the comparison between 2009 earthquake catastrophe and COVID pandemic has been made, relevant changes of ICU doctors in professional role (from anesthesiologist to resuscitation specialist), team, environment, shifts, and work organization are evidenced.

These doctors, who already experienced the 2009 earthquake reported a feeling of greater insecurity facing this latter catastrophe, i.e., the COVID pandemic, as well as perception of greater concern for their family and the global situation. Most of them, on the other hand, believed that the organization of the current crisis was better, and their ability to cope with the emergency greater.

The relevant differences in the characteristics of the two emergencies must be considered in terms of the longer duration of the COVID19 pandemic, the kind of exposure to the danger and the threat to personal and family safety.

Narratively, the doctors involved in this study reported that having directly participated in the medical management of another emergency (the 2009 earthquake) appears to have contributed to limiting demoralization and psychological distress, as a kind of “vaccine effect.”

Possible explanation is that a stronger feeling of participation in the organization of work represents a barrier to burnout. As a matter of fact, when we studied psychological distress along participation in work organization, we found less perception and symptoms in those most involved.

Active participation in work organization appears to act as a protective factor from anxiety, as a persistent fear response to a stressful environmental condition, even if the COVID-19 pandemic has had a disruptive effect on ICUs by highlighting critical deficiencies. After years of bed and staff containment the health emergency situation demonstrated the limitations of the intensive care structure and led to a rapid restructuring. The ICU structure in Italy is designed for managing medical emergencies in “normal” incidence and prevalence situations. Exceptional emergencies such as those due to natural disasters, wars, collective trauma, pandemic, and need quantitatively sufficient health facilities at the same time elastic to adapt to major requests and to everyday live also considering the costs. Bearing in mind the differences that in terms of the influx of patients over time, and the typology of clinical problems, from a qualitative point of view, the fundamental need is good organization, and the motivation of the ICU health professionals. The significant observation of a cluster of worse responses to psychological assessment is evident in those professionals who considered themselves less involved in the organization of work. If so, the feeling of having decision-making possibilities and greater participation in the organization of work is crucial for the health and wellbeing of professionals themselves and strengthen coping skills in the face of an emergency.

### Limitations of the study

Some limitations need to be considered. The study was conducted at a single center so that generalization is limited. The sample is undoubtedly small, but it included all healthcare professionals working in the ICU. This relationship can be considered a case study, a research approach of heuristic value, which could provide hypotheses that can be further tested in a larger sample.

The relatively small sample size does not allow further subgrouping. Important subgrouping is by gender: Our sample is mainly composed of women (84% of the total sample), probably with a greater perceptive capacity of emotional reactions to stressors (Tarsitani and Biondi, 1999; Mandarelli et al., 2004). We used a basic psychological assessment and performed correlations among the variables considered without a control sample.

## Conclusion

The COVID-19 outbreak has placed extraordinary demands on health systems around the world, putting a strain on the wellbeing of Italian health workers.

Targeted interventions on health professionals, constantly assessing stress levels, improving coping strategies, by provision of psychological counseling and adequate training, are essentials to make them less vulnerable, reduce the stress level and the risk of developing anxiety and depression (Dal’Bosco et al., 2020; Koulong and Xuemei, 2020). This is especially important for ICU professionals, who have a higher workload and more direct contact with patients in extreme, life-threatening situations (Zerbini et al., 2020).

Management of strong emotions and stress, satisfaction of basic needs, effective and efficient communication, equal distribution of tasks, flexible working hours and psychological support without stigmatization can be elements of these interventions. Increasing protective variables, such as resilience and personal fulfillment, is vital for improving preventative measures at work, particularly for professionals who have high emotional exhaustion and depersonalization scores as risk factors (Lourdes et al., 2020).

The introduction of a psychological screening, either for selection or monitoring of the mental health of ICU professionals, especially if they are working during a health crisis such as the current one can be therefore suitable.

These results can be, however considered from a heuristic point of view. These issues need to be addressed in further investigations with a larger sample. This is the object of our ongoing research.

## Data availability statement

The raw data supporting the conclusions of this article will be made available by the authors, without undue reservation.

## Ethics statement

The studies involving human participants were reviewed and approved by Local Ethic Committee L’Aquila Italy. The patients/participants provided their written informed consent to participate in this study.

## Author contributions

CL, PS, and AlP contributed to the analysis and interpretation of data. AnP, AC, IM, and FM contributed to drafting the article. All authors contributed to the article and approved the submitted version.
